# Brain-gut-liver axis: Chronic psychological stress promotes liver injury and fibrosis *via* gut in rats

**DOI:** 10.3389/fcimb.2022.1040749

**Published:** 2022-12-12

**Authors:** Meng-Yang Xu, Can-Can Guo, Meng-Ying Li, Yu-Han Lou, Zhuo-Ran Chen, Bo-Wei Liu, Ling Lan

**Affiliations:** ^1^ Department of Gastroenterology and Hepatology, the First Affiliated Hospital of Henan University, Kaifeng, China; ^2^ Department of Infectious Diseases, Jining No.1 People′s Hospital, Jining, China; ^3^ Department of Gastroenterology and Hepatology, Kaifeng Central Hospital, Kaifeng, China; ^4^ Department of Gastroenterology and Hepatology, Henan Provincial People’s Hospital, People’s Hospital of Zhengzhou University, People’s Hospital of Henan University, Zhengzhou, China; ^5^ Department of Gastroenterology and Hepatology, Henan No.3 Provincial People’s Hospital, Zhengzhou, China

**Keywords:** gut microbiota, lipopolysaccharide, brain-gut axis, gut-liver axis, psychological stress, liver injury, liver fibrosis

## Abstract

**Background:**

The effect of chronic psychological stress on hepatitis and liver fibrosis is concerned. However, its mechanism remains unclear. We investigated the effect and mechanism of chronic psychological stress in promoting liver injury and fibrosis through gut.

**Methods:**

Sixty male SD rats were randomly assigned to 6 groups. Rat models of chronic psychological stress (4 weeks) and liver fibrosis (8 weeks) were established. The diversity of gut microbiota in intestinal feces, permeability of intestinal mucosa, pathologies of intestinal and liver tissues, collagen fibers, protein expressions of toll-like receptor 4 (TLR4), myeloid differentiation factor 88 (MyD88), nuclear factor kappa β (NF-κβ), tumor necrosis factor α (TNF-α) and interleukin 1 (IL-1) in liver tissue, liver function and coagulation function in blood and lipopolysaccharide (LPS) in portal vein blood were detected and analyzed.

**Results:**

The diversities and abundances of gut microbiota were significant differences in rats among each group. The pathological lesions of intestinal and liver tissues, decreased expression of occludin protein in intestinal mucosa, deposition of collagen fibers and increased protein expression of TLR4, MyD88, NF-κβ, TNF-α and IL-1 in liver tissue, increased LPS level in portal vein blood, and abnormalities of liver function and coagulation function, were observed in rats exposed to chronic psychological stress or liver fibrosis. There were significant differences with normal rats. When the dual intervention factors of chronic psychological stress and liver fibrosis were superimposed, the above indicators were further aggravated.

**Conclusion:**

Chronic psychological stress promotes liver injury and fibrosis, depending on changes in the diversity of gut microbiota and increased intestinal permeability caused by psychological stress, LPS that enters liver and acts on TLR4, and active LPS-TLR4 pathway depend on MyD88. It demonstrates the possibility of existence of brain-gut-liver axis.

## 1 Background

Liver fibrosis is an inevitable stage in various chronic liver diseases that progress into cirrhosis. Due to the protracted course, and even uncontrollable progression to liver cirrhosis, liver fibrosis causes heavy mental pressure and psychological burden to many patients. Some studies have shown that the proportion of patients with liver fibrosis and cirrhosis who experience psychological disorders, such as depression and anxiety, is as high as 50%, which in turn seriously affects the therapeutic effect of liver disease and the quality of life in patients (Bajaj et al., 2017). Over recent years, the effect of chronic psychological stress on exacerbation of hepatitis and progression of liver fibrosis has been acknowledged as a relevant factor (Ishtiaq et al., 2018; Soto-Angona et al., 2020). However, its mechanism remains unclear.

Progressive liver disease is often accompanied by gut microbial imbalance, intestinal endotoxemia and bacterial translocation (Nolan, 2010; Macnaughtan and Jalan, 2015; Shim and Jeong, 2020; Yang X. et al., 2020; Park et al., 2022). Lipopolysaccharide (LPS), the main component of endotoxin, can activate intracellular signal transduction by the use of toll-like receptor 4 (TLR4) in the liver, thus exacerbating liver inflammation, and promoting the progression of liver fibrosis, which is the “gut-liver” axis (Seki et al., 2007; Szabo, 2015; Tilg et al., 2020; Mandato et al., 2021; Rao et al., 2021; T Wang et al., 2022). Besides, an interactive effect between psychological stress and the gut has also been confirmed by the studies in rats, which is the “brain-gut” axis (Stasi et al., 2012; Arotcarena et al., 2020; Hulme et al., 2020; Deng et al., 2021). We hypothesized that chronic psychological stress might affect the liver *via* gut. By assessing gut microbiota, intestinal permeability, bacterial translocation, liver injury and liver fibrosis in rats, this study explored the effect and mechanism of chronic psychological stress in causing liver injury and promoting liver fibrosis.

## 2 Materials and methods

### 2.1 Animals

Studies were performed on male SD rats (10-12 weeks, 300-400g) obtained from Henan Animal Experiment Center. Rats were housed in an environment with a temperature of 22 ± 1°C, a relative humidity of 50 ± 1% and a light/dark cycle of 12/12 hr. Rats were fed with regular diet. This study was performed in line with the principles of the Declaration of Helsinki. Approval was granted by the Ethics Committee of Zhengzhou University.

### 2.2 Grouping and modeling of rats

The sample size was calculated by a formal power analysis. The significance level of α was set to 0.05. Fisher’s exact test showed that the sample size of each group was at least 6, which can provide > 80% potency to detect the differences between groups. Rats were randomized into 6 groups: 1) normal group (*n* = 6): no modeling; 2) olive oil group (oil group, *n* = 8): subcutaneous injection of olive oil was administrated at 3ml/kg (the initial dose was doubled), 2 times/week for 8 weeks; 3) chronic psychological stress group (stress group, *n* = 8): normal rats were subjected to selecting 10 unpredictable stimulating factors, namely by chronic unpredictable stress method, for 4 weeks from the 5th week onwards; 4) olive oil+psychological stress group (oil+stress group, *n* = 8): chronic psychological stress was induced for 4 weeks from the 5th week of olive oil model; 5) liver fibrosis group (fibrosis group, *n* = 15): 40% CCl4 was subcutaneously administrated, and the dose and time were the same as those in the oil group; 6) liver fibrosis+chronic psychological stress group (fibrosis+stress group, *n* = 20): chronic psychological stress was induced for 4 weeks from the 5th week of liver fibrosis model.

Rats were sacrificed after 8 weeks of modeling. Blood was taken from the portal vein for immediate submission. Intestinal feces were stored at -80°C. Intestinal and liver tissues were fixed with 4% paraformaldehyde or stored at -80°C.

### 2.3 Establishment of rat model of chronic psychological stress

Chronic unpredictable stress method was used to create a chronic psychological stress model, namely by selecting 10 unpredictable stimulating factors to stimulate the rats (Nirmal et al., 2008; Chudzik et al., 2022): 1) damp padding plus tilt feeding: the cage was tilted by 30° to soak the bottom padding with water for 24h; 2) water fasting for 24h; 3) food fasting for 24h; 4) electric shock: electrical stimulation with biological functional system was performed with current of 0.1mA at 3 times/min for 15min; 5) behavioral restraint for 2h: After anesthesia by intraperitoneal injection with sodium pentobarbital (10mg/ml) at a dose of 1.5ml/kg, rats were in the overhead position and tied on a special rat plate, and timing was started after rats were awakened; 6) unfamiliar objects: beakers, barbed wire, etc. were randomly selected and placed in the cage for 24 h; 7) alternate day and night: the cages were covered with black cloth for 12 h during the day, and irradiated with incandescent lamps for 12h after night time; 8) unfamiliar smell: medical cotton dipped with glacial acetic acid was placed into open glass bottle, which was put in the cage for 24h; 9) swimming in the hot water: rats were put in the water with a temperature of 48°C and a depth of 35 cm and were let there to swim for 10 min; 10) swimming in the ice water: rats were put in the water with a temperature of 4°C and a depth of 35cm and were let there to swim for 10 min. One of the 10 stimulating methods was randomly selected every day, and the same was never repeated for two consecutive days. The stimulating method was changed every day at 9 am for a total of 4 weeks.

### 2.4 HE staining of liver and intestinal tissues

Paraffin sections of liver and intestinal tissues were prepared and dewaxed to water; hematoxylin staining was performed for 5 min and differentiated with 1% hydrochloric acid ethanol solution for 30 s. HE staining was performed for 1-2min with gradient ethanol dehydration; xylene was used for transparentizing, neutral gum for mounting, and the pathological changes were observed under a light microscope.

### 2.5 Masson staining of liver tissue

Paraffin sections of liver tissue were stained with Masson trichrome, and collagen fibers in liver tissue were observed under an optical microscope. The degree of liver fibrosis through semi-quantitative staging was scored as follows: 0 points (no liver fibrosis), no collagen fibers; 1 point (mild liver fibrosis), collagen fibers were located in the central hepatic lobule; 2 points (moderate liver fibrosis), collagen fibers spread along the central hepatic lobule; 3 points (severe liver fibrosis), collagen fibers extended to the edge of the hepatic lobule; 4 points (cirrhosis), formation of pseudolobule.

### 2.6 Detection of liver function and coagulation function in blood

The automatic biochemical analyzer was used for the detection of alanine aminotransferase (ALT), aspartate aminotransferase (AST), total bilirubin (TBIL) in serum, and prothrombin time (PT) and PT activity (PT%) in whole blood.

### 2.7 Azo chromogenic LAL assay of LPS in portal vein blood

Portal vein plasma was pretreated by a modified perchloric acid method, and the LPS concentration was determined by referring to the instructions of the chromogenic LAL assay.

### 2.8 16S rRNA sequencing analysis of gut microbiota

The E.Z.N.A.^®^ Soil DNA Kit (Omega Bio-tek Corporation, USA) was used to extract rat fecal genomic DNA. The high-fidelity DNA FastPfu Polymeras (Transgen Company, China) was adopted to conduct PCR amplification in the V3-V4 variable region of the 16S rRNA gene. The used primers were as follows: 338F (5’-ACTCCTACGGGAGGCAGCAG-3’) and 806R (5’-GGACTACHVGGGTWTCTAAT-3’). PCR reaction conditions: 95°C for 3min; 95°C for 30s, 55°C for 30s, 72°C for 45s, with 27 cycles in total; 72°C for 10min. There were 3 PCR repeats for each sample. The three repetitive PCR products were mixed. The PCR products were extracted using a 2% agarose gel. The recovered products were purified using the AxyPrep DNA Gel Extraction Kit (Axygen, USA). The library was constructed using the the NEXTFLEX Rapid DNA-Seq Kit (Bioo Scientific, USA), and sequencing was made with the MiSeq Reagent Kit v (Illumina, USA) using the Miseq PE300 platform (Illumina, USA). UPARSE software (version 7.1, http://drive5.com/uparse/) was used to perform OTU clustering on sequences based on 97% similarity. The table of OUT abundance was obtained and used for the bioinformatics analysis of gut microbiota diversity.

### 2.9 Evaluation of the degree of intestinal mucosal lesions

The evaluation of the degree of intestinal mucosal lesions was performed as follows: 1) three complete intestinal mucosal villi were randomly selected in each section, and their length and width were measured. The average values were used as intestinal mucosal morphological parameters. 2) the degree of intestinal mucosal damage was evaluated with the following scoring criteria (intestinal mucosal damage index): 0 points, intestinal mucosal villi were normal; 1 point, villous subepithelial space and capillary were congested; 2 points, enlarged subepithelial space, moderate edema in the lamina propria, and dilated central lacteals; 3 points, obvious edema of the lamina propria, degeneration and necrosis of intestinal mucosal epithelial cells, and exfoliation of a few villus tips; 4 points, degeneration, necrosis, and exfoliation of epithelial cell layers, exfoliation of part of villous tissues, exposure of lamina propria, dilation and congestion of capillaries; 5 points, villous exfoliation, disintegration of the lamina propria, bleeding or ulceration.

### 2.10 Immunohistochemical staining of occludin protein in intestinal tissue

Immunohistochemical staining of intestinal tissue was performed using the rabbit anti-occludin monoclonal antibody (Abcam, UK) (1:250) and horseradish peroxidase-labeled anti-rabbit antibody. Image-Pro plus 6.0 image analysis software was used to calculate the integral optical density (IOD) of positive staining for semi-quantification of occludin protein expression.

### 2.11 Immunohistochemical staining and western blot of TLR4 in liver tissue

Immunohistochemical staining of liver tissue was performed using the rabbit anti-TLR4 polyclonal antibody (Abcam, UK) (1:200) and horseradish peroxidase-labeled anti-rabbit antibody. Western blot of the protein of liver tissue was performed using the rabbit anti-TLR4 polyclonal antibody (Abcam, UK) (1: 1000) and mouse anti-β-actin monoclonal antibody (Abcam, UK) (1: 5000). Gel-pro 4.0 electrophoresis analysis software was used to measure IOD for relative quantification of TLR4 protein expression.

### 2.12 Immunohistochemical staining of signaling proteins in LPS-TLR4 pathway depend on MyD88 in liver tissue

Immunohistochemical staining of liver tissue was performed using the rabbit anti-MyD88/NF-κβ/TNF-α/IL-1 polyclonal antibodies (Abcam, UK) (1:250) and horseradish peroxidase-labeled anti-rabbit antibody. Image-Pro plus 6.0 image analysis software was used to calculate IOD for semi-quantifications of these protein expressions.

### 2.13 Statistical analysis

SPSS 25.0 software was used for statistical analysis. The measurement data were presented as mean ± SD. ANOVA was used for homogeneous test of variance. LSD (variance was homogeneous) or Tamhane T_2_ (variance was not homogeneous) test was used for comparison between two groups. For the analysis of Beta diversity, a distance matrix was calculated based on unweighted unifrac distance. Principal coordinate analysis (PCoA) was used to visualize the results, and Adonis analysis was used to detect whether the differences between groups were significant and the degree of explanation for the differences between groups by grouping factors. Using STAMP software, the Kruskal-Wallis rank-sum test was used to analyze the differences in flora abundance among multiple groups. The Wilcoxon rank-sum test was used to compare the two groups. Two-tailed testing was used for all statistical tests. *p <* 0.05 was considered statistically significant.

## 3 Results

### 3.1 General conditions

Compared with normal rats, rats exposed to chronic psychological stress had disordered and dull fur, reduced activity and food intake, listlessness, and weight loss; rats with liver fibrosis had more significant symptoms mentioned above; rats with liver fibrosis manifested the most severe symptoms after receiving chronic psychological stress, while some developed ascites. Among the 65 enrolled rats, 7 died during the modeling (2 in the fibrosis group and 5 in the fibrosis+stress group), and 58 were included in the analysis.

### 3.2 Pathological changes of liver tissue

**Figure 1:**
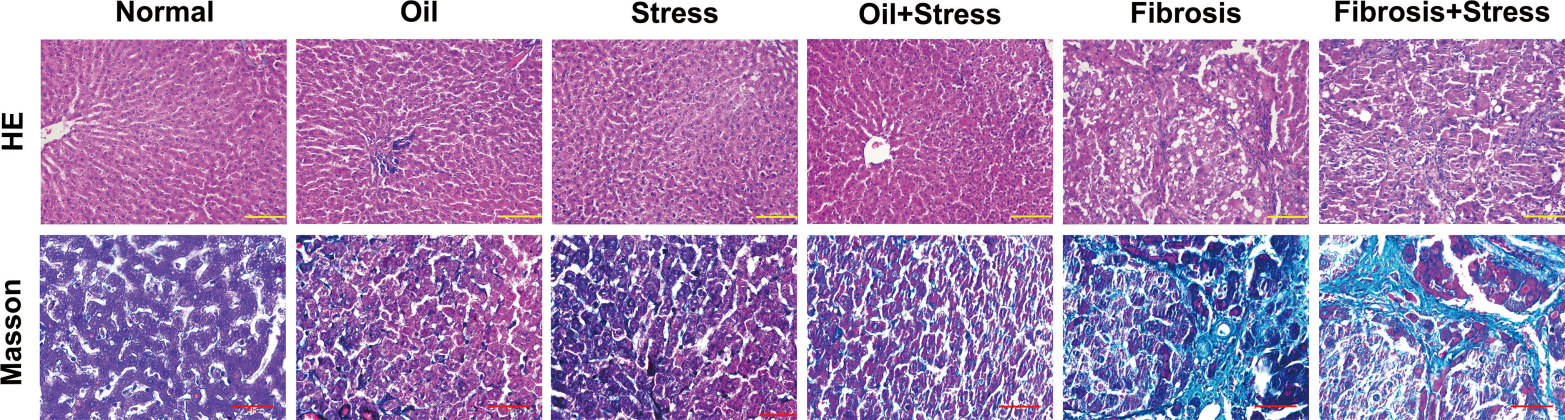
HE and Masson staining of liver tissue in rats. **(up)** HE staining of liver tissue. The degeneration and necrosis of hepatocytes and inflammatory cell infiltration occurred in liver tissue in the stress group and oil+Stress group, and were further exacerbated in the fibrosis group and fibrosis+stress group. Scale bar: 100um. **(down)** Masson staining of collagen (green) in liver tissue. Very small collagen fibers formed in liver tissue in the stress group and oil+stress group, numerous collagen fibers were deposited in liver tissue in the fibrosis group and fibrosis+stress group. Scale bar: 50um.

Degeneration and necrosis of hepatocytes and inflammatory cell infiltration occurred in the liver tissue of rats exposed to chronic psychological stress. Liver tissue of rats with liver fibrosis showed extensive and severe fatty degeneration and necrosis of hepatocytes; infiltration of inflammatory cells was present around the portal and central veins; the portal area was enlarged, and proliferation of numerous fibrous tissues was found. After chronic psychological stress was applied to rats with liver fibrosis, degeneration and necrosis of hepatocytes, infiltration of inflammatory cells and proliferation of fibrous tissue were further exacerbated (**Figure 1** up).

### 3.3 Collagen formation and fibrosis staging in liver tissue

Masson staining (**Figure 1** down) and liver fibrosis staging (**Table 1**) showed that very small collagen fibers formed in the liver tissue of rats exposed to chronic psychological stress, which was mild; there was no statistical difference in scores of liver fibrosis staging compared with normal rats (0.13 ± 0.35 *vs.* 0 ± 0, *p* > 0.05). For rats with liver fibrosis, numerous collagen fibers were formed in the liver tissue, and fibrosis staging was significantly higher (2.46 ± 0.88) compared to normal rats (*p* < 0.05). Under the effect of chronic psychological stress for rats with liver fibrosis, the collagen fibers of liver tissue were deposited in large amounts, and the formation of the fibrous septum was partially seen. The score of fibrosis staging was further increased (2.93 ± 0.70) compared with the liver fibrosis group, but the statistical difference was not significant (*p* > 0.05).

**Table 1:** Scores of hepatic fibrosis staging by Masson staining.

**Group**	***n***	**Stage (*n*)**					**Score** **(means ± SD)**
		0	1	2	3	4	
**Normal**	6	6	0	0	0	0	0 ± 0
**Oil**	8	8	0	0	0	0	0 ± 0
**Stress**	8	7	1	0	0	0	0.13 ± 0.35
**Oil+Stress**	8	7	1	0	0	0	0.13 ± 0.35
**Fibrosis**	13	0	1	5	6	1	2.46 ± 0.88^*†^
**Fibrosis+Stress**	15	0	0	4	8	3	2.93 ± 0.70^*†^

^*^p < 0.05 vs. normal group and oil groups; ^†^p < 0.05 vs. stress group, oil group and oil+stree groups.

### 3.4 Liver function and coagulation function

Serum ALT level in rats exposed to chronic psychological stress were increased, and significantly different compared with normal rats (*p* = 0.016); the levels of AST, TBIL, PT and PT% did not significantly change (*p* > 0.05). The levels of ALT, AST, TBIL, PT and PT% in rats with liver fibrosis were significantly higher than those in normal rats (*p* < 0.001). After chronic psychological stress was applied to rats with liver fibrosis, liver function and coagulation function were significantly abnormal. Although there was no significant difference compared to the liver fibrosis group (*p* > 0.05), the levels of the five indicators showed further exacerbation (**Table 2**).

**Table 2:** Liver function and coagulation function of rats (means ± SD).

Group	*n*	ALT(U/L)	AST(U/L)	TBIL(μmol/L)	PT(s)	PT%(%)
normal	6	40.33 ± 6.47	123.00 ± 16.59	0.90 ± 0.14	16.33 ± 0.63	66.33 ± 4.41
oil	8	44.13 ± 7.90	141.13 ± 29.87	1.29 ± 0.57	15.81 ± 0.61	69.50 ± 4.87
stress	8	110.88 ± 38.19^*^	349.38 ± 158.17	1.05 ± 0.28	17.04 ± 1.01	57.75 ± 6.23
oil+stress	8	106.38 ± 36.80^*^	328.75 ± 177.91	1.14 ± 0.31	17.14 ± 1.03	58.38 ± 6.80
fibrosis	13	232.92 ± 66.72^*†^	468.31 ± 122.43^*^	4.35 ± 0.98^*†^	21.27 ± 2.65^*†^	44.15 ± 7.95^*†^
fibrosis+stress	15	273.60 ± 127.26^*†^	558.47 ± 226.16^*^	6.69 ± 2.98^*†^	22.31 ± 3.37^*†^	42.13 ± 8.85^*†^

^*^p < 0.05 vs. normal group and oil groups; ^†^p < 0.05 vs. stress group and oil+stress groups. ALT, alanine aminotransferase; AST, aspartate aminotransferase; TBIL, total bilirubin; PT, prothrombin time; PT%, PT activity.

### 3.5 LPS levels in portal vein blood

Compared with the normal group (18.89 ± 2.61 EU/L) and the oil group (21.79 ± 2.41 EU/L), the LPS levels of the portal vein in the liver fibrosis group were significantly increased (99.73 ± 10.30 EU/L, *p* < 0.001), and the stress (35.03 ± 4.78 EU/L, *p* < 0.001) and oil+stress (37.64 ± 5.68 EU/L, *p* = 0.001) groups also showed increases, but not as significant as the liver fibrosis group. The LPS levels of the portal vein in the fibrosis+stress group (122.17 ± 13.21 EU/L) were further increased compared to the liver fibrosis group; the observed difference was statistically significant (*p* < 0.001).

### 3.6 Diversity and abundance of gut microbiota

**Figure 2:**
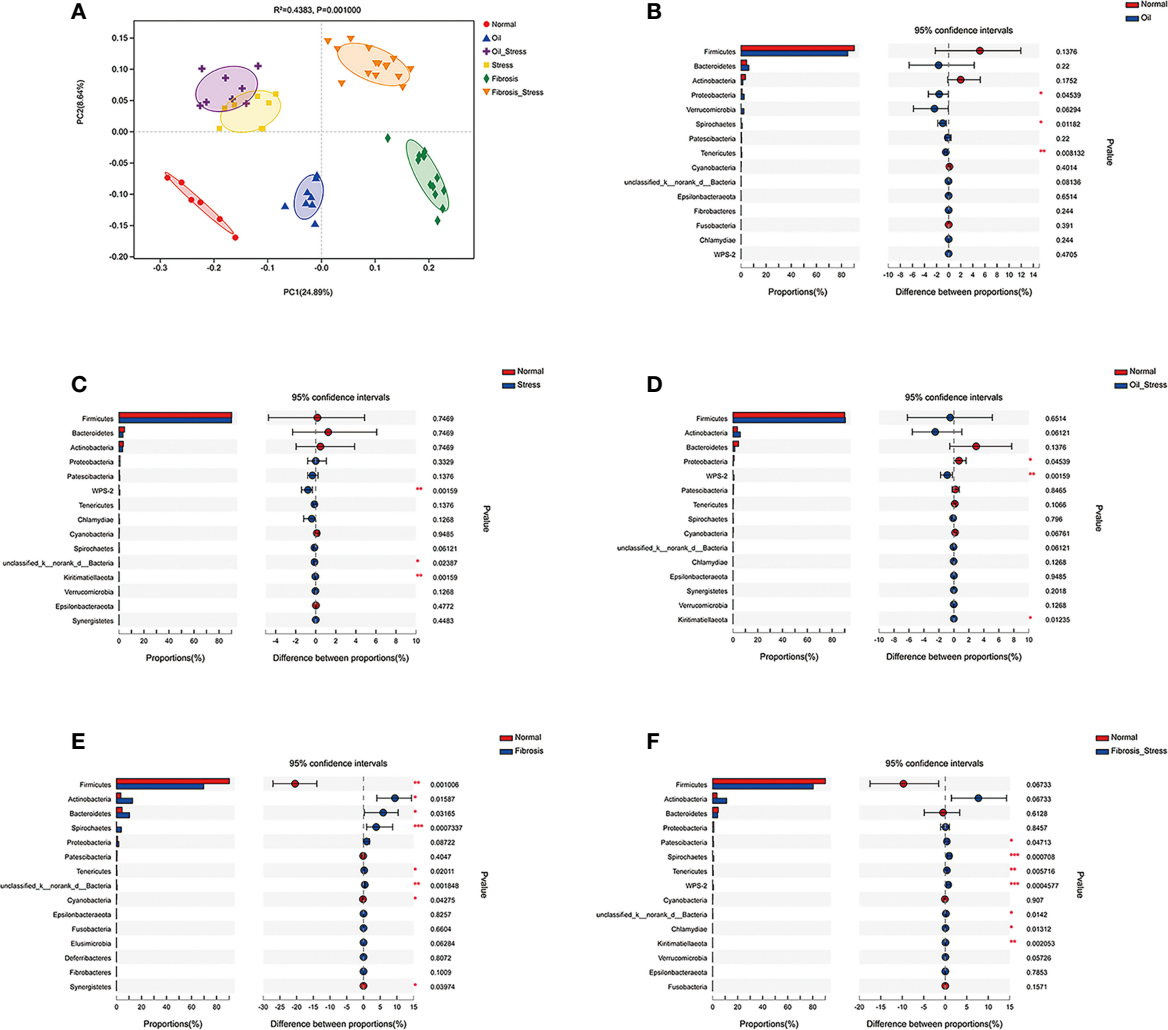
Diversity of gut microbiota in rats. **(A)** PCoA analysis of Beta diversity of gut microbiota. **(B-F)** Difference analysis of the abundance of gut microbiota at the phylum level vs. the normal group. **(B)** *Proteobacteria* was increased in the oil group (*p* < 0.05). **(C)** *WPS-2 bacteria* was increased in the stress group (*p* < 0.01). **(D)** *Proteobacteria* (*p* < 0.05) and *WPS-2 bacteria* (*p*<0.01) were increased in the oil+stress group. **(E)** *Firmicutes* (*p* < 0.01), *Actinobacteria* (*p*<0.05), *Spirochaete* (*p* < 0.01), *Tenericutes* (*p* < 0.05) and *Bacteroidetes* (*p* < 0.05) were increased in the fibrosis group. **(F)** *WPS-2 bacteria* (*p* < 0.01), *Firmicutes* (*p* > 0.05), *Actinobacteria* (*p* > 0.05), *Spirochaete* (*p* < 0.01), *Tenericutes* (*p* < 0.01), *Patescibacteria* (*p* < 0.05) and *WPS-2 bacteria* (*p* < 0.001) were increased in the fibrosis+stress group. Data are shown as means ± SD. *p < 0.05; **p<0.01; ***p < 0.001 vs. normal group.

PCoA analysis showed that olive oil, psychological stress, and liver fibrosis all caused shifts in the beta diversity of rat gut microbiota to varying degrees (**Figure 2A**). The analysis of abundance of gut microbiota at the phylum level showed that compared with the normal group, the injection of olive oil resulted in an increased abundance of *Proteobacteria* in rats (**Figure 2B**, *p =* 0.045; **Figure 2D**, *p =* 0.045). Chronic psychological stress significantly increased the abundance of *WPS-2 bacteria* in the intestine of rats (**Figure 2C**, *p =* 0.002; **Figure 2D**, *p =* 0.002; **Figure 2F**, *p =* 0.001). Liver fibrosis led to decreased abundance of intestinal *Firmicutes* (**Figure 2E**, *p =* 0.001; **Figure 2F**, *p =* 0.068), and increased abundances of *Actinobacteria* (**Figure 2E**, *p =* 0.016; **Figure 2F**, *p =* 0.068), *Spirochaete* (**Figure 2E**, *p =* 0.001; **Figure 2F**, *p =* 0.001) and *Tenericutes* (**Figure 2E**, *p =* 0.020; **Figure 2F**, *p =* 0.006). In addition, the abundance of *Bacteroidetes* was increased in the liver fibrosis group (**Figure 2E**, *p =* 0.032); the abundances of *Patescibacteria* (*p =* 0.047) and *WPS-2 bacteria* (*p* < 0.001) were significantly increased in the fibrosis + stress group (**Figure 2F**).

### 3.7 Pathological changes and mucosal lesions of intestinal tissue

**Figure 3:**
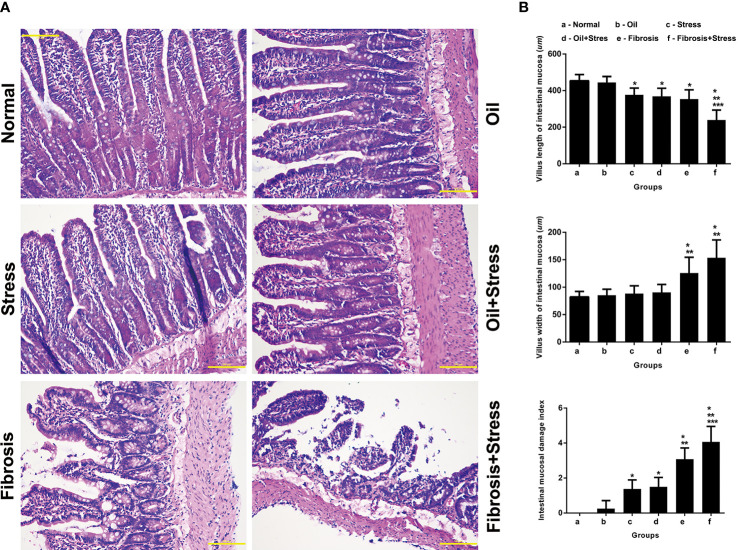
Mucosal lesions of intestinal tissue in rats. **(A)** HE staining of intestinal tissue. Scale bar: 100 um. **(B)** Quantification of mucosal lesions in intestinal tissue. **(up)** Villus length of intestinal mucosa. **(middle)** Villus width of intestinal mucosa. **(down)** Intestinal mucosal damage index. Data are shown as means ± SD. *p < 0.05 vs. normal group and oil groups; **p < 0.01 vs. stress group and oil+stree groups; ***p < 0.001 vs. fibrosis group.

The intestinal villi were sparsely arranged in rats exposured to chronic psychological stress, and the epithelial cells were disorderly arranged, and the sizes were different (**Figure 3A**). The villus length was significantly shorter than in normal rats (**Figure 3B** up, *p* = 0.024). The intestinal mucosal damage index was significantly higher than that of normal rats (**Figure 3B** down, *p* = 0.002).

The number of intestinal villi, which became shorter and thicker, was decreased in rats with liver fibrosis, the shape was irregular, and part of the villi were broken. The disordered arrangement and different size of epithelial cells, degeneration, necrosis, and disintegration of lamina propria were observed (**Figure 3A**). The length and width of villi were significantly shorter or thicker than those in normal rats (**Figure 3B** up, *p* = 0.002; **Figure 3B** middle, *p =* 0.002), and the width were significantly thicker than those with chronic psychological stress (**Figure 3B** middle, *p =* 0.012). The intestinal mucosal damage was significantly worse compared with normal rats and those with chronic psychological stress (**Figure 3B** down, *p* < 0.001).

After rats with liver fibrosis were exposed to chronic psychological stress, changes in the number and morphology of intestinal villi, and degeneration and necrosis of epithelial cells were further exacerbated. The villi were thicker and shorter, and mucosal damage was significantly worse (**Figure 3A**). Compared with rats with liver fibrosis, there were statistical differences in the villi length (**Figure 3B** up, *p* < 0.001) and mucosal damage index (**Figure 3B** down, *p* = 0.031). Compared with rats exposed to chronic psychological stress, (**Figure 3B** up, *p* < 0.001; **Figure 3B** down, *p* = 0.031), there were statistical differences in the length and width of villi and mucosal damage index (**Figure 3B**, *p* < 0.001).

### 3.8 Occludin protein expression in intestinal tissue

**Figure 4:**
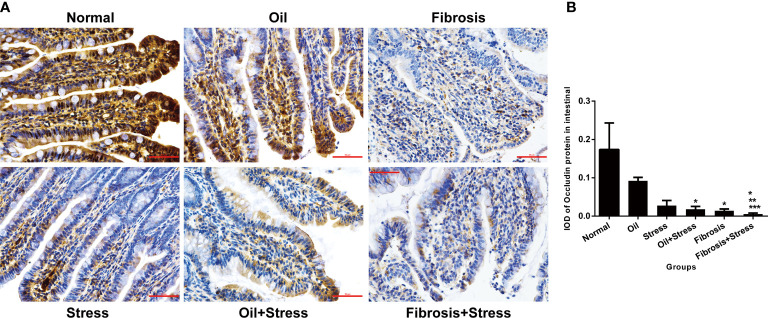
Occludin protein expression in intestinal tissue of rats. **(A)** Immunohistochemical staining of occludin protein in intestinal tissue. Scale bar: 50um. **(B)** Semi-quantitative analysis of occludin protein in intestinal tissue. Data are shown as means ± SD. *p < 0.05 vs. normal group and oil groups; **p < 0.01 vs. stress group and oil+stress groups; ***p < 0.001 vs. fibrosis group.

Occludin protein was abundantly expressed in intestinal tissue of normal rats, and its expression was significantly decreased in both the stress group (*p* = 0.047) and the liver fibrosis group (*p* = 0.035), but there was not statistically different between both groups (*p =* 0.31). After rats with liver fibrosis were exposed to chronic psychological stress, occludin protein expression was further reduced compared with rats with liver fibrosis and those with chronic psychological stress (*p* < 0.001) (**Figure 4**).

### 3.9 TLR4 protein expressions in liver tissue

**Figure 5:**
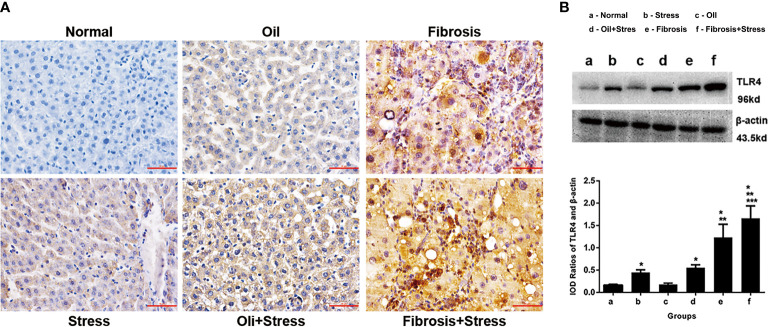
TLR4 protein expression in liver tissue of rats. **(A)** Immunohistochemical staining of TLR4 protein in liver tissue. Scale bar: 50um. **(B)** Western blot and quantitative analysis of TLR4 protein in liver tissue. **(up)** The typical image of TLR4 protein expression. **(down)** Quantification of TLR4 protein expression (IOD). Data are shown as means ± SD. *p < 0.05 vs. normal group and oil groups; **p < 0.01 vs. stress group and oil+stree groups; ***p < 0.001 vs. fibrosis group. TLR4, toll-like receptor 4; IOD, integral optical density.

The immunohistochemical results showed that TLR4 protein was weakly expressed in liver tissue of normal rats, and TLR4 protein expression was increased in rats exposed to chronic psychological stress. TLR4 protein expression in the liver fibrosis group was significantly increased, and after rats with liver fibrosis were exposed to chronic psychological stress, TLR4 protein expression was further increased compared with the liver fibrosis group (**Figure 5A**).

The western blot result showed that compared with normal rats, TLR4 protein expression in liver tissue of rats exposed to chronic psychological stress was increased (*p* < 0.001). TLR4 protein expression in rats with liver fibrosis was further increased compared to those exposed to chronic psychological stress (*p* < 0.001). For rats with liver fibrosis exposed to chronic psychological stress, the TLR4 expression level was more significantly increased, and significantly different (*p* = 0.008), compared with rats with liver fibrosis and those with chronic psychological stress (**Figure 5B**).

### 3.10 Signaling protein expressions in LPS-TLR4 pathway depend on MyD88 in liver tissue

**Figure 6:**
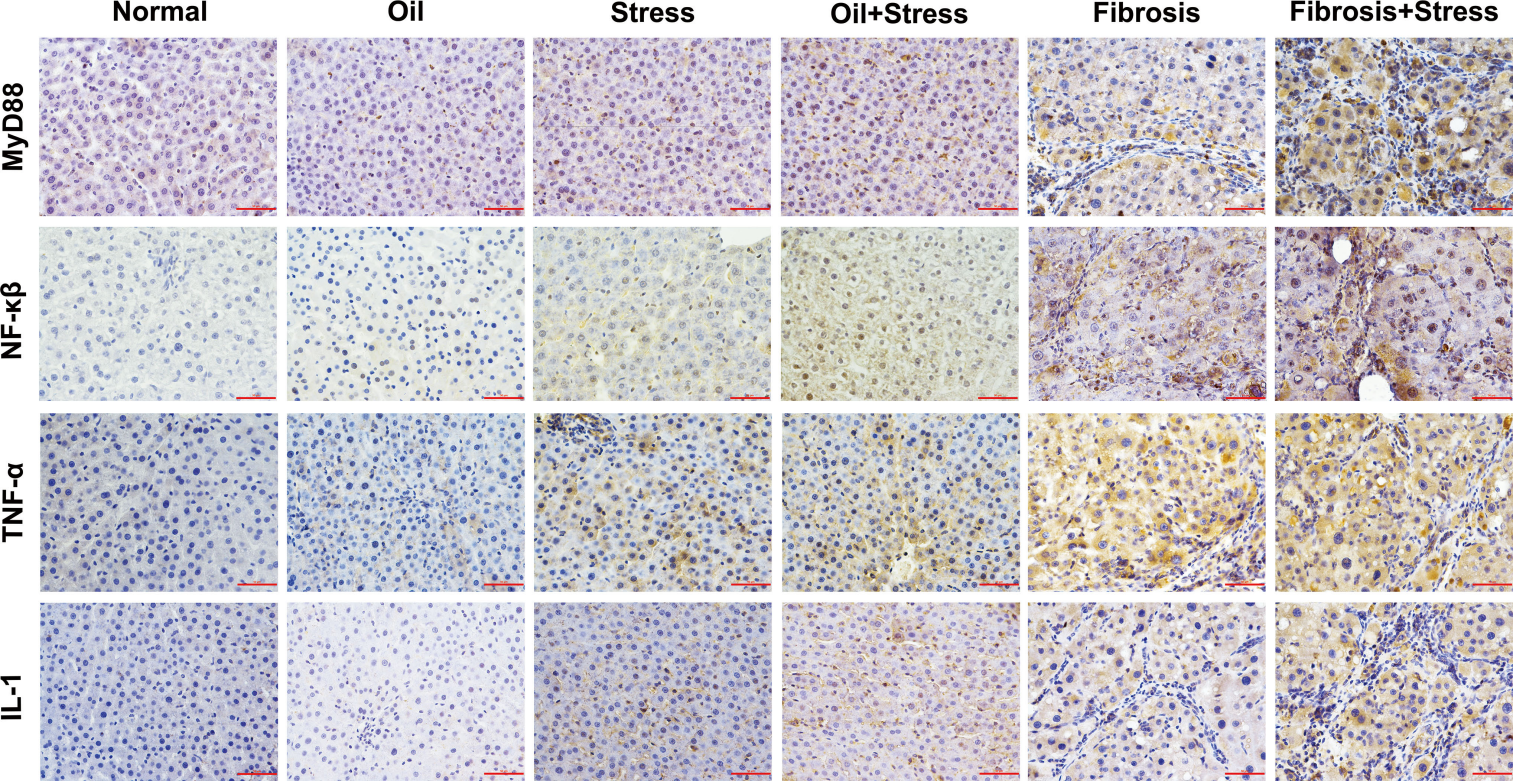
Immunohistochemical staining of MyD88, NF-κβ, TNF-α and IL-1 proteins in LPS-TLR4 pathway depend on MyD88 in liver tissue of rats. Scale bar: 50um. MyD88, myeloid differentiation factor 88; NF-κβ, nuclear factor kappa β; TNF-α, tumor necrosis factor α; IL-1, interleukin 1.

The immunohistochemical results (**Figure 6**) and IOD analysis (**Table 3**) showed that the proteins of MyD88, NF-κβ TNF-α and IL-1, which were the intracellular downstream signal proteins in LPS-TLR4 pathway depend on MyD88, weakly expressed in liver tissue of normal rats, and their expressions were increased in rats exposed to chronic psychological stress. These protein expressions in the liver fibrosis group were significantly increased, and after rats with liver fibrosis were exposed to chronic psychological stress, their expressions were further increased compared with the liver fibrosis group.

**Table 3:** Semi-quantitative analysis (IOD) of signaling protein expressions in LPS-TLR4 pathway depend on MyD88 in liver tissue (means ± SD).

Group	*n*	IOD (×10^-3^)			
		MyD88	NF-κβ	TNF-α	IL-1
normal	6	0.66 ± 0.14	0.006 ± 0.002	0.27 ± 0.16	0.03 ± 0.009
oil	8	1.45 ± 0.20	1.03 ± 0.21	0.91 ± 0.18	0.82 ± 0.15
stress	8	4.7 ± 0.17^*^	3.62 ± 0.60^*^	11.21 ± 0.42^*^	6.41 ± 0.48a^*^
oil+stress	8	10.48 ± 0.23^*^	10.85 ± 2.09^*^	12.48 ± 1.89^*^	9.86 ± 0.48^*^
fibrosis	13	18.10 ± 2.17^*†^	22.71 ± 1.18^*†^	30.93 ± 1.01^*†^	12.71 ± 1.30^*†^
fibrosis+stress	15	52.31 ± 3.39^*†‡^	33.47 ± 1.66^*†‡^	41.35 ± 3.38^*†‡^	23.66 ± 2.18^*†‡^

*p < 0.05 vs. normal group and oil groups;^†^p < 0.05 vs. stress group and oil+stree groups; ^‡^p < 0.05 vs. fibrosis group. IOD, integral optical density; MyD88, myeloid differentiation factor 88; NF-κβ, nuclear factor kappa β; TNF-α, tumor necrosis factor α; IL-1, interleukin 1.

## 4 Discussion

Recently, the concern about the relationship between chronic stress and chronic disease is increasing (Crielaard et al., 2021). Chronic psychological stress is considered as one of the factors involved in the progression and worsening of hepatitis, liver fibrosis, cirrhosis, and even liver cancer (Reichel et al., 2018). In the present study, we found that chronic psychological stress alone could induce necrosis of hepatocytes, inflammation of liver tissue, and liver function damage in rats. Rats with liver fibrosis exposed to chronic psychological stress had a more significant inflammatory response of the liver. Although deposition of collagen fibers and abnormalities in liver and coagulation functions were not significantly different from those in rats with liver fibrosis, there was a trend of further exacerbation. And interestingly, the rats with liver fibrosis manifested the most severe symptoms after experiencing chronic psychological stress, which indicated the superposition of double factors. Liver damage might lead to decreased tolerance to psychological stress. We will observe external indicators that reflect the degree of psychological disorder of rats, such as sugar water experiment, maze experiment, etc, and further analyze in the future study.

Chronic psychological stress has a definite effect on the liver. However, its underlying mechanisms and inter-organic signaling pathways remain unknown. Most studies have suggested that chronic psychological stress affects the liver in a direct way. Chronic psychological stress activates HPA axis and releases glucocorticoids (GCs) that enter the liver through circulation. It induces liver inflammation, accelerates cell apoptosis and reduces liver blood flow leading to the progression of liver fibrosis (Yoneda et al., 2002; Petrescu et al., 2018). Nevertheless, other studies have found that an increased GCs in the body under psychological stress can lead to an increased Fas antigen (a key factor in inducing apoptosis) in liver and accelerate apoptosis. However, if liver cells are cultured with GCs *in vitro*, Fas expression does not increase (Chida et al., 2004). This suggests that under psychological stress *in vivo*, the effect of GCs on liver is not achieved through direct action, and there may be more complicated indirect pathways (Chida et al., 2004; de Sousa Rodrigues et al., 2017). It is possible that the liver is affected through indirect effects or intermediaries.

In progressive liver diseases, such as steatohepatitis, liver fibrosis and cirrhosis, even liver cancer, proliferation and disorder of gut microbiota may occur due to weakened intestinal motility and reduced secretion of bile salt, intestinal permeability may be increased due to declines in intestinal immune function, and bacterial translocation can occur (Fouts et al., 2012; Lee et al., 2020; Wang and Chen, 2020; Yang X. et al., 2020; Behary et al., 2021; Yan et al., 2021; Forlano et al., 2022; Park et al., 2022). Intestinal bacteria and endotoxin (mainly composed of LPS) are translocated to mesenteric lymph nodes or other extraintestinal parts, where they bind to LPS binding protein (LBP) with transport function, and then transport LPS to the cell membrane to bind to CDl4, thus forming the LPS-LBP-CDl4 complex. This complex then enters the liver through the circulation of the portal vein, triggering a cascade of intracellular signals in liver through TLR4, which eventually leads to exacerbated liver fibrosis (Seki et al., 2007), cirrhosis (Milosevic et al., 2019; Wang et al., 2022) and even liver cancer (Meng et al., 2018). This interaction effect between the gut microbiota and the liver mediated by the LPS-TLR4 signaling pathway is called the gut-liver axis (Seki et al., 2007; Chassaing et al., 2014; Cheng et al., 2022).

Under stress, the central nervous system (CNS) of brain can release corticotropin releasing factor (CRF), and CRF can also be released in the intestine (Kiank et al., 2010). Both of them can act on the intestinal nervous system (ENS) of the intestine, activating CRF receptor 1 (CRFR1) (o'malley et al., 2010) on the ENS, so as to regulate intestinal functions, such as intestinal secretion, sensation, dynamics, neuropeptide and hormone release, immune and inflammatory responses, and bacterial balance. This is called the brain-gut axis (Rhee et al., 2009; Bailey et al., 2011). Furthermore, CNS can regulate intestinal functions through some neuropeptides such as 5-HT, γ-aminobutyric acid, norepinephrine and dopamine, etc. Numerous studies have confirmed that excessive or harmful chronic psychological stress can cause intestinal dysfunction, increased mucosal permeability, gut dysbiosis, microbial imbalance and bacterial translocation (Rhee et al., 2009; Stasi et al., 2012; Forsythe et al., 2016; Sundman et al., 2017; Martin et al., 2018; Dalton et al., 2019; Jacobs and Mayer, 2019; Deng et al., 2021; Li et al., 2022). It indicates the intersection of the brain-gut axis and gut-liver axis is intestinal bacterial translocation (Chudzik et al., 2022). Therefore, it is hypothesized that chronic psychological stress causes translocation of intestinal bacteria and LPS through the brain-gut axis. Then LPS acts on TLR4 in liver through the “gut-liver” axis, activating intracellular downstream signaling pathways, and ultimately contributing to liver inflammation and progression of liver fibrosis. In the present study, we observed the gut microbiota, intestinal mucosa pathology, LPS in portal vein blood, and TLR4 in liver tissue, which were selected to evaluate whether chronic psychological stress may affect the liver through the intestine, thus causing liver injury and promoting liver fibrosis.

Intestinal barriers, which can protect against many diseases, include a mechanical barrier, chemical barrier, immune barrier, and biological barrier (Citi, 2018). Among them, mechanical and biological barriers are the most important. The main component of the mechanical barrier is the tight junction complex, which is formed by the interaction between 4 trans-membrane proteins (occludin, claudins, junction adhesion molecule, and tricellulin) with zonula occludens proteins (ZO1, ZO2, and ZO3), where occludin protein has an important role in protecting intestinal barrier function and regulating intestinal permeability (Clark and Mach, 2016). The intestinal biological barrier is mainly composed of intestinal resident bacteria, 99% of which are obligate anaerobes, such as *Bacteroides* and *Firmicutes*. The gut microbiota provides protection and nutrition to the intestinal mucosa, and form a barrier against excessive proliferation and infection of exogenous antigens (Cani, 2018).

Previous studies have shown that psychological stress can lead to damage of intestinal mucosa and imbalance of gut microbiota, resulting in down-regulated expression of intestinal occludin protein, increased intestinal permeability and intestinal bacterial translocation (Clark and Mach, 2016; Sundman et al., 2017). This is consistent with the results of the present study that revealed the lesions of intestinal mucosal, decreased occludin protein and changes in diversity and abundance of gut microbiota. The disorder of gut microbiota caused by the changes of diversity, quantity, activity and distribution of gut microbiota (Nishida et al., 2018; Rao et al., 2021). The results of the present study showed that the species and proportion of gut microbiota in normal rats were similar with that in human body. Injection of olive oil, liver fibrosis and chronic psychological stress could independently or jointly cause the changes of gut microbiota diversity and abundance. Olive oil injection increased the abundance of *Proteobacteria* in rats, which is Gram-negative bacteria, accounting for less than 1% of gut microbiota, including *Salmonella*, *Pseudomonas aeruginosa* and other pathogenic bacteria. This suggests that although olive oil injection is not enough to cause liver damage and fibrosis (it is usually used as a negative control for rat model with liver fibrosis), it already can cause changes in gut microbiota and increased number of pathogenic bacteria.

The present study also showed a decreased abundance of *Firmicutes*, and increased abundances of *Bacteroidetes, Actinobacteria*, *Spirochaete* and *Tenericutes* in rats with liver fibrosis. *Firmicutes* is the most main gut microbiota of human. Its subordinate *Lactobacillus* are beneficial bacteria, which can participate in intestinal metabolism, enhance intestinal immune function, and inhibit the growth of harmful bacteria. *Bacteroidetes* is the second largest gut microbiota of human, which has a symbiotic relationship with *Firmicutes*, and the ratio of the two can be used as one of the effective indicators to judge the imbalance of gut microbiota. *Actinobacteria* is Gram-positive bacteria. Its subordinate *Bifidobacteria* are intestinal beneficial bacteria, while other subordinate bacteria are mostly pathogenic bacteria, such as *Mycobacterium tuberculosis* and *Mycobacterium leprae*. *Spirochaete* and *Tenericutes* are few in the normal human intestine tract, but many of their subordinate bacteria are harmful bacteria which can mediate the inflammatory reaction of the intestine and have potential pathogenicity. The other study also found that there was a disorder of gut microbiota in patients with liver cirrhosis, through the quantitative analysis of the microflora in the feces, which was characterized by a decrease in the proportions of dominant microbiota including *Firmicutes* and *Bacteroidetes*, and an increase in harmful bacteria such as *Enterococcus Enterobacteriaceae* (Bajaj et al., 2014).

We also found that chronic psychological stress caused a significant increase in the abundance of *WPS-2 bacteria*. The two factors of liver fibrosis and psychological stress had synergistic effect, which led to a decrease in the abundance of *Firmicutes*, and an increase of *Bacteroidetes*, *Actinobacteria Spirochaete*, *Tenericutes*, WPS-2 bacteria and *Patescibacteria*. *WPS-2 bacteria*, recently proposed as Candidatus eremiobacterota, has been found in the gut and mouth of plants and mammals in recent years. It is related to photosynthesis in plants, but its characteristics in mammals are rarely studied (Ward et al., 2019). *Patescibacteria* is usually detected in the anaerobic environment, which may involve the anaerobic metabolism of the body (Qiu et al., 2020). However, it has been found that the number of *Enterobacter faecalis* and *Escherichia coli* in the gut microbiota of anxiety rats increased (Jang et al., 2018). Another population-based survey shows that mental patients are accompanied by gut microbiota disorder. For example, patients with depression often have an increase abundance of *Bacteroidetes* (Qin et al., 2010; Yang J. et al., 2020). *Proteobacteria and Actinobacteria* (Qin et al., 2010) and a decreased abundance of *Firmicutes* (Qiu et al., 2020), *Blautia* and *Eubacterium* (Yang J. et al., 2020). These results of previous studies are partly consistent with our results, but also different. In particular, the increase of the abundance of *WPS-2 bacteria* and *Patescibacteria* is the first discovery in the present study, which is worth further study and investigation.

Interesting, the changed gut microbiota, increased pathogenic bacteria and mild decreased expression of occludin protein were found in the oil group compared with the normal group. We think that the lipid composition and subcutaneous absorption of olive oil might cause irritation to the abdominal cavity and intestine, which did not rule out slight adverse effects on the liver. We will add other negative controls for comparative analysis in the future study.

The direct consequence of impaired intestinal barrier function is that harmful substances, such as LPS, enter into the circulatory system (Forlano et al., 2022). LPS can cause disorders to the immune system within the liver *via* “gut-liver” axis and mediate the inflammatory cascade, up-regulate Fas antigen expression in liver cells, activate Kupffer cells and hepatic stellate cells, thereby accelerating liver apoptosis, promoting the formation of the extracellular matrix, leading to liver injury and progression of liver fibrosis (Muschen et al., 1998; Henao-Mejia et al., 2012; Kumar et al., 2017; Castillo-Dela Cruz et al., 2019; Hu et al., 2020; Hu et al., 2021; Forlano et al., 2022). Additionally, some studies have found that anxiety can also increase LPS levels in feces and blood of rats (Jang et al., 2018). Our results showed that chronic psychological stress could indeed increase LPS levels in portal vein blood of rats, and lead to further elevation of LPS levels in portal vein blood of rats with liver fibrosis. This suggests that chronic psychological stress can cause intestinal bacterial translocation and may affect the liver through the “gut-liver” axis *via* the portal vein, thus, inducing liver injury and potentially promoting the progression of liver fibrosis.

TLR4 can identify LPS, which is a key regulator of the host’s inflammatory response (Xue et al., 2015; Lee et al., 2017; Metwally et al., 2020). TLR4 is mainly located in Kupffer cells, hepatic stellate cells and hepatocytes in liver. Activated TLR4 pathway can exacerbate liver inflammation and fibrosis by secreting pro-inflammatory cytokines, activating hepatic stellate cells and generating extracellular matrixes (Seki and Brenner, 2008). Previous studies have shown that TLR4 in fibrotic liver tissue is increased, and positively correlated with the degree of liver fibrosis (Hua et al., 2007). Our results revealed that under the effect of chronic psychological stress or liver fibrosis modeling, the expression level of TLR4 protein in liver tissue of rats could be increased, while in rats with liver fibrosis exposed to chronic psychological stress, TLR4 protein expression of liver tissue was further increased. This suggested that chronic psychological stress was involved in the activation of the TLR4 signaling pathway in the liver, which may damage the liver and promote the progression of liver fibrosis through the TLR4 signaling pathway.

LPS-TLR4 pathway involves an intracellular downstream signal transduction pathway in liver that is called the MyD88 dependent pathway. This signal pathway is mediated by MyD88 to activate transcription NF-κβ (Huang et al., 2014), and then promote downstream gene transcriptions of TNF-α, IL-1 and IL-6, etc. (Roh and Seki, 2013). MyD88 is an important junction protein in LPS-TLR4 pathway, which can combine with proteins or other signal molecules to induce downstream signal transduction (Kogut et al., 2005). During the formation of liver fibrosis, the protein expression of MyD88 in hepatic stellate cells increased significantly, suggesting that MyD88 plays an important role on liver fibrosis (Adachi et al., 1998). The present study also showed that both liver fibrosis and chronic psychological stress could lead to LPS-TLR4 pathway depend on MyD88 be activated.

## 5 Conclusions

**Figure 7:**
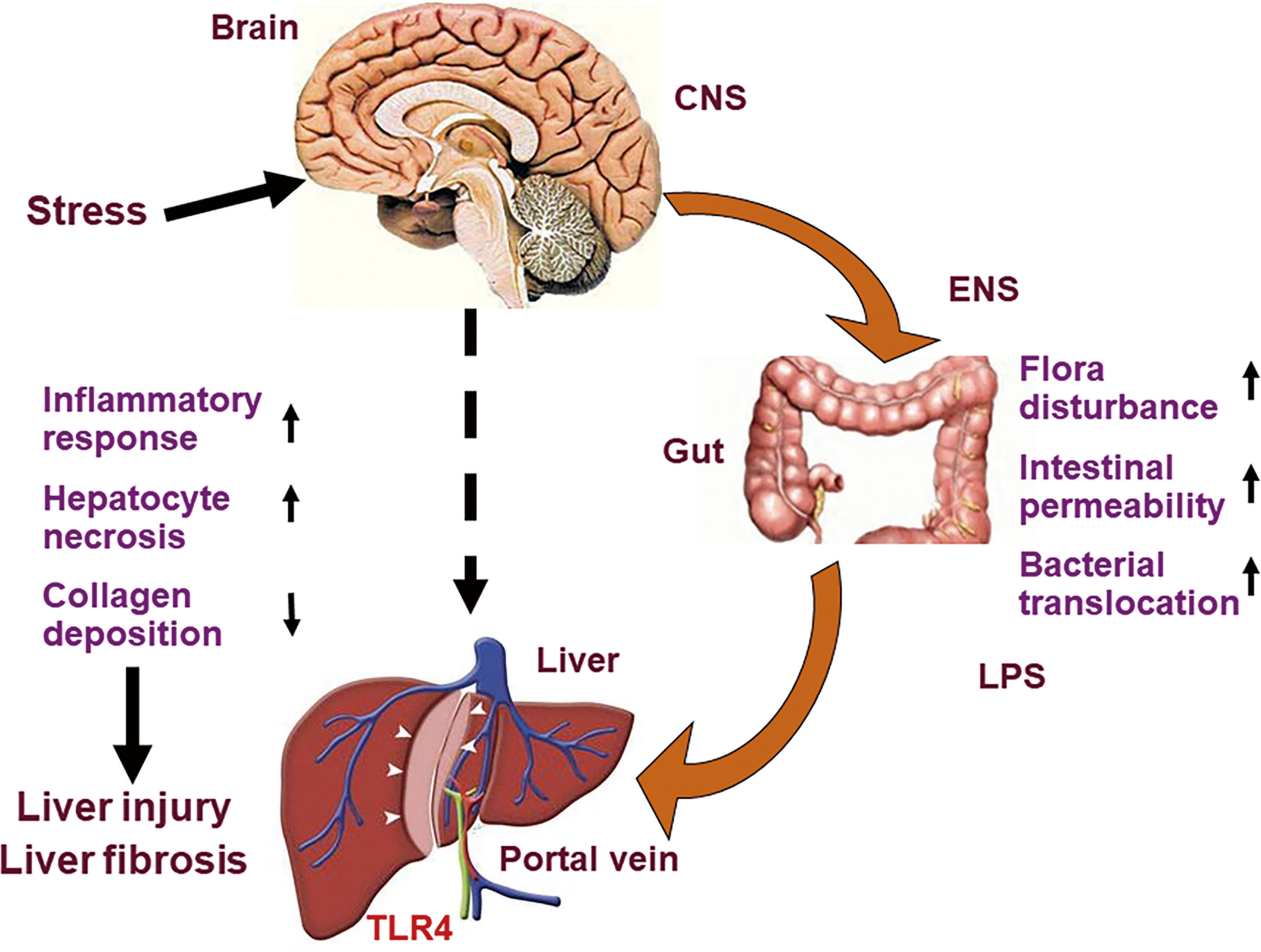
Diagram of brain-gut-liver axis. Chronic psychological stress induces changes of gut microbiota, increased intestinal permeability, bacterial translocation and increased LPS of portal vein blood, then triggers LPS-TLR4 pathway in liver, and promotes liver injury and fibrosis. LPS, lipopolysaccharide; TLR4, toll-like receptor 4.

Chronic psychological stress can be used as an independent risk factor to cause liver injury in rats and as a potential exacerbating factor to promote the progression of liver fibrosis. This may be related to the fact that chronic psychological stress can induce changes of gut microbiota, increased intestinal permeability, bacterial translocation and increased LPS of portal vein blood, then trigger LPS-TLR4 pathway in liver (**Figure 7**). We put forward a pathway of brain-gut-liver axis, which might illuminate the mechanism underlying liver injury and fibrosis caused by chronic psychological stress. However, whether the benefits of relieving liver damage and improving intestinal mucosal barrier status can be obtained, by some interventions, including intestinal microbiota regulating measures, liver protective drugs and blocking the brain-gut axis etc.? Further study should be conducted and analyzed by the above interventions for confirming the brain-gut-liver axis.

## Data availability statement

The datasets presented in this study can be found in online repositories. The names of the repository/repositories and accession number(s) can be found below: https://www.ncbi.nlm.nih.gov/sra/PRJNA884024. 

## Ethics statement

The animal study was reviewed and approved by Ethics Committee of Zhengzhou University.

## Author contributions

Conceptualization, LL. Methodology, M-YX, C-CG, M-YL and B-WL. Formal Analysis, Z-RC and Y-HL. Writing – Original Draft Preparation, M-YX, C-CG and M-YL. Writing – Review & Editing, LL. Supervision, LL. Funding Acquisition, LL. All authors contributed to the article and approved the submitted version.
